# The peroxisome proliferator-activated receptor gamma (PPARγ) agonist, rosiglitazone, ameliorates neurofunctional and neuroinflammatory abnormalities in a rat model of Gulf War Illness

**DOI:** 10.1371/journal.pone.0242427

**Published:** 2020-11-13

**Authors:** Kaspar Keledjian, Orest Tsymbalyuk, Stephen Semick, Mitchell Moyer, Serban Negoita, Kevin Kim, Svetlana Ivanova, Volodymyr Gerzanich, J. Marc Simard

**Affiliations:** 1 Department of Neurosurgery, University of Maryland School of Medicine, Baltimore, MD, United States of America; 2 Department of Pathology, University of Maryland School of Medicine, Baltimore, MD, United States of America; 3 Department of Physiology, University of Maryland School of Medicine, Baltimore, MD, United States of America; 4 Neurosurgical Service, Veterans Affairs Maryland Health Care System, Baltimore, MD, United States of America; Fu Jen Catholic University, TAIWAN

## Abstract

**Background:**

Gulf War (GW) Illness (GWI) is a debilitating condition with a complex constellation of immune, endocrine and neurological symptoms, including cognitive impairment, anxiety and depression. We studied a novel model of GWI based on 3 known common GW exposures (GWE): (i) intranasal lipopolysaccharide, to which personnel were exposed during desert sand storms; (ii) pyridostigmine bromide, used as prophylaxis against chemical warfare; and (iii) chronic unpredictable stress, an inescapable element of war. We used this model to evaluate prophylactic treatment with the PPARγ agonist, rosiglitazone (ROSI).

**Methods:**

Rats were subjected to the three GWE for 33 days. In series 1 and 2, male and female GWE-rats were compared to naïve rats. In series 3, male rats with GWE were randomly assigned to prophylactic treatment with ROSI (GWE-ROSI) or vehicle. After the 33-day exposures, three neurofunctional domains were evaluated: cognition (novel object recognition), anxiety-like behaviors (elevated plus maze, open field) and depression-like behaviors (coat state, sucrose preference, splash test, tail suspension and forced swim). Brains were analyzed for astrocytic and microglial activation and neuroinflammation (GFAP, Iba1, tumor necrosis factor and translocator protein). Neurofunctional data from rats with similar exposures were pooled into 3 groups: naïve, GWE and GWE-ROSI.

**Results:**

Compared to naïve rats, GWE-rats showed significant abnormalities in the three neurofunctional domains, along with significant neuroinflammation in amygdala and hippocampus. There were no differences between males and females with GWE. GWE-ROSI rats showed significant attenuation of neuroinflammation and of some of the neurofunctional abnormalities.

**Conclusion:**

This novel GWI model recapitulates critical neurofunctional abnormalities reported by Veterans with GWI. Concurrent prophylactic treatment with ROSI was beneficial in this model.

## Introduction

Gulf War (GW) Illness (GWI) is a chronic disorder affecting 25–40% of the nearly 700,000 men and women who served in the 1990–1991 Persian Gulf War [1, 2]. GWI is characterized by a variety of systemic symptoms as well as symptoms referable to specific organs. Arguably, the most debilitating symptoms are those that affect the central nervous system (CNS), including sleep disturbances, difficulties with thinking, concentration and memory, and anxiety and depression [1, 3]. Neuropsychological and cognitive problems that persist more than a decade remain a dominant complaint [4, 5]. CNS involvement and neurodegeneration in GWI have been corroborated by structural and functional neuroimaging and biomarker studies [3, 6–12].

The factors responsible for GWI are still under active investigation. Epidemiological studies suggest possible links between GWI and significant exposure to one or more GW-related chemicals, including pyridostigmine bromide pills, neurotoxicants (sarin, cyclosarin and diisopropyl fluorophosphate nerve agents, and various organophosphate-based and pyrethroid-based insecticides), and the smoke from oil fires and “burn pits” [1, 2, 13, 14]. Several animal studies examining effects on neuroinflammation or neurofunction have modeled GWI based on exposures to various combinations of GW-related chemicals [15–24].

Apart from GW-related chemicals, deployed men and women also were exposed to sand-dust particles (SDP) from frequent sand storms, which predisposed them to upper respiratory and small airway diseases [25, 26]. SDP play an important role in Al Eskan disease, which overlaps clinically with GWI [27, 28]. SDP contain a variety of chemical and microbiological materials, including airborne ultrafine particles, particulate matter, and lipopolysaccharide (LPS), which cause upper respiratory and small airway inflammation [29–32]. Notably, compared to samples from Europe, sand from the Middle East (Jordan) contains remarkably high levels of 3-hydroxy acids of 10, 12, and 14 carbon chain lengths, which are specific markers of LPS [33]. Intranasal LPS alone is known to induce cytokine expression within the CNS and to lead to depression-like behaviors [34]. To date, however, animal studies modeling GWI have not incorporated intranasal LPS.

Physiological stress experienced in theater also has been proposed as a potential contributor to GWI [35]. Exposure to GW-related chemicals and SDP likely was compounded by chronic unpredictable stress (war fighting, extreme temperatures, sleep deprivation, physical exertion) [1]. Among personnel who were in Iraq or Kuwait, where all the battles took place, GWI was most strongly associated with using pyridostigmine bromide pills and being within one mile of an exploding SCUD missile [36]. Chronic unpredictable stress is a major factor of war that can have lasting consequences [37]. Several animal studies examining effects on neuroinflammation or neurofunction have modeled GWI based on exposures to both GW-related chemicals and various stressors, including the administration of corticosterone [24, 38–41] or restraint or other physical stressor [42–51].

Animal studies of GWI have repeatedly shown evidence of neuroinflammation, which is known to lead to neurodegeneration [52]. Animal models with exposures to GW-related chemicals alone, as well as models with combined exposures to GW-related chemicals and stressors, report neuroinflammation in various brain regions, as assessed by a variety of cytokines and chemokines, including many downstream of nuclear factor κB (NF-κB) [22, 24, 38–41, 50]. A characteristic feature of neuroinflammation is glial activation. With few exceptions [17, 18, 38, 39, 47], most models of GWI have shown robust glial activation involving both astrocytes [16, 18, 22, 44, 48, 50] and microglia [22, 48, 50]. In GWI models, treatments that reduce neuroinflammation have been found to improve neurofunctional abnormalities [22, 48, 51]. Recently, the first direct evidence of neuroinflammation in vivo in humans with GWI was reported, based on PET imaging for the glial inflammatory marker, translocator protein (TSPO) [53].

Peroxisome proliferator activated receptors (PPAR) are ligand-activated transcription factors with three isoforms. α, β/δ and γ, that play important roles in regulating glucose absorption, homeostasis of lipid metabolism, cell growth and differentiation, and repressing the expression of pro-inflammatory genes [54–58]. Thiazolidinediones such as rosiglitazone and pioglitazone are potent agonists of PPARγ that induce significant neuroprotection in animal models of CNS disease [54]. In glial cells and macrophages, PPARγ activation leads to the inhibition of pro-inflammatory gene expression, due to silencing of transcription factors such as activator protein-1 (AP-1), STAT1 and NF-κB. PPARγ also mediates down-regulation of pro-inflammatory genes such as cyclooxygenase-2 (COX-2), metalloproteinase-9 (MMP-9), scavenger receptor A, inducible nitric oxide synthase (NOS2), as well as many pro-inflammatory cytokines, chemokines, and interleukins, many of which have been identified in various models of GWI. PPARγ agonists have been shown to attenuate neuroinflammation in several neuroinflammatory settings, including TSPO upregulation [59, 60]. To date, however, PPARγ agonists have not been evaluated in animal models of GWI.

Here, we studied a novel rat model of GWI induced by a combination of three plausible GW-related exposures: oral pyridostigmine, intranasal LPS and unpredictable stress. Using this model, we evaluated the effects of the PPARγ agonist, rosiglitazone, on neurofunctional abnormalities and neuroinflammation.

## Materials and methods

### Ethics statement

We certify that all applicable institutional and governmental regulations concerning the ethical use of animals were followed during the course of this research. Animal experiments were performed under a protocol approved by the Institutional Animal Care and Use Committee (IACUC) of the University of Maryland School of Medicine and the Baltimore Veterans Affairs Hospital, and in accordance with the relevant guidelines and regulations as stipulated in the United States National Institutes of Health Guide for the Care and Use of Laboratory Animals. All efforts were made to minimize the number of animals used and their suffering. Experiments are reported in compliance with the ARRIVE guidelines.

### Subjects and study protocol

Wistar rats, 12–15-weeks old (Harlan/Envigo, Indianapolis, IN), were used for this study. The 33-day GWE consisted of the following: (1) the drinking water contained pyridostigmine bromide (PYR) (Sigma Aldrich), 40 mg/L; drinking 10 mL/day delivered ~1.3 mg/kg/day [61, 62]; (2) under mild isoflurane anesthesia, LPS (Sigma Aldrich; 50 μL of a 10 mg/5 mL solution) was pipetted into the nasal cavity once daily, alternating sides, 5 days per week; (3) chronic unpredictable stress (CUS) was administered daily, as described below.

We studied 3 consecutive series of rats. *Series 1* consisted of 11 naïve male rats with no exposures and 12 male rats with the 33-day GWE. *Series 2* consisted of 3 naïve male rats with no exposures, 7 male rats with the 33-day GWE and 7 female rats with the 33-day GWE. *Series 3* consisted of 20 rats, all with the 33-day GWE, randomly assigned to receive vehicle (GWE-VEH group; n = 10) or rosiglitazone (GWE-ROSI group; n = 10).

Rosiglitazone maleate (ROSI) (Abcam, Cambridge, MA) was prepared by dissolving 1 gm in 10 mL dimethyl sulfoxide (DMSO), aliquoted and kept at –20°C for daily use. Rats in the GWE-ROSI group received ROSI (3 mg/rat), administered daily in 50% maple syrup (0.2 mL) for 33 days, while rats in the control groups (GWE-VEH) received only maple syrup; each animal was given their daily oral dose individually by allowing them to suck the maple syrup containing the rosiglitazone from a 1 mL syringe, as described [63].

### Chronic unpredictable stress (CUS)

The CUS procedure was adapted from previous studies [64–68]. The CUS procedure involved the daily application of various stressors for a period of 33 days. The stressors were scheduled in a pseudorandom fashion over a 33-day period, as shown in Table 1. The various stressors included: (a) restraint; rats were kept individually in closed ventilated tubes (21×7×7 cm diameter) (Harvard Apparatus, Holliston, MA) for 4 hours; (b) cold room; rats were kept in a refrigerated cold room (5°C) in their original cages for 3–4 hours; (c) wet bedding: 500 mL water was added to the bedding for 3–4 hours; (d) food deprivation: food was removed for up to 36 hours; (e) water deprivation: rats were deprived of water for up to 36 hours; (f) day/night reversal and isolation: rats were isolated in individual cages and left in a lighted room overnight; (g) cold water swimming: rats were forced to swim in 6–8°C water for up to 4–5 minutes; (h) crowded shaking: 3 rats were placed in a mouse cage on a shaking platform for 30 minutes; (i) restraint/tail pinch: rats were restrained as above and a pinch clamp was placed at a distance of about 3 cm from the base of the tail for 10 mins. Non-stressed animals were left undisturbed in their home cages except during housekeeping procedures such as cage cleaning and weighing.

**Table 1:** List of stressors and day when applied.

Stressor	Day applied
Restraint	1, 9, 24,29
Cold Room	2, 10, 14, 28,
Wet Bedding	3, 8, 21, 27,
Food Deprivation	4,5, 18,19, 32, 33
Water Deprivation	11, 12, 25, 26
Isolation and Lights On overnight	6, 16, 23, 30
Swimming at 6–8°C	7, 15, 22, 31
Crowded Shaking	16, 20, 23, 30
Restraint/Tail Pinch	13, 20
Intranasal LPS	weekdays
Pyridostigmine Bromide *per orem*	daily

### Neurofunctional tests

**Fig 1:**
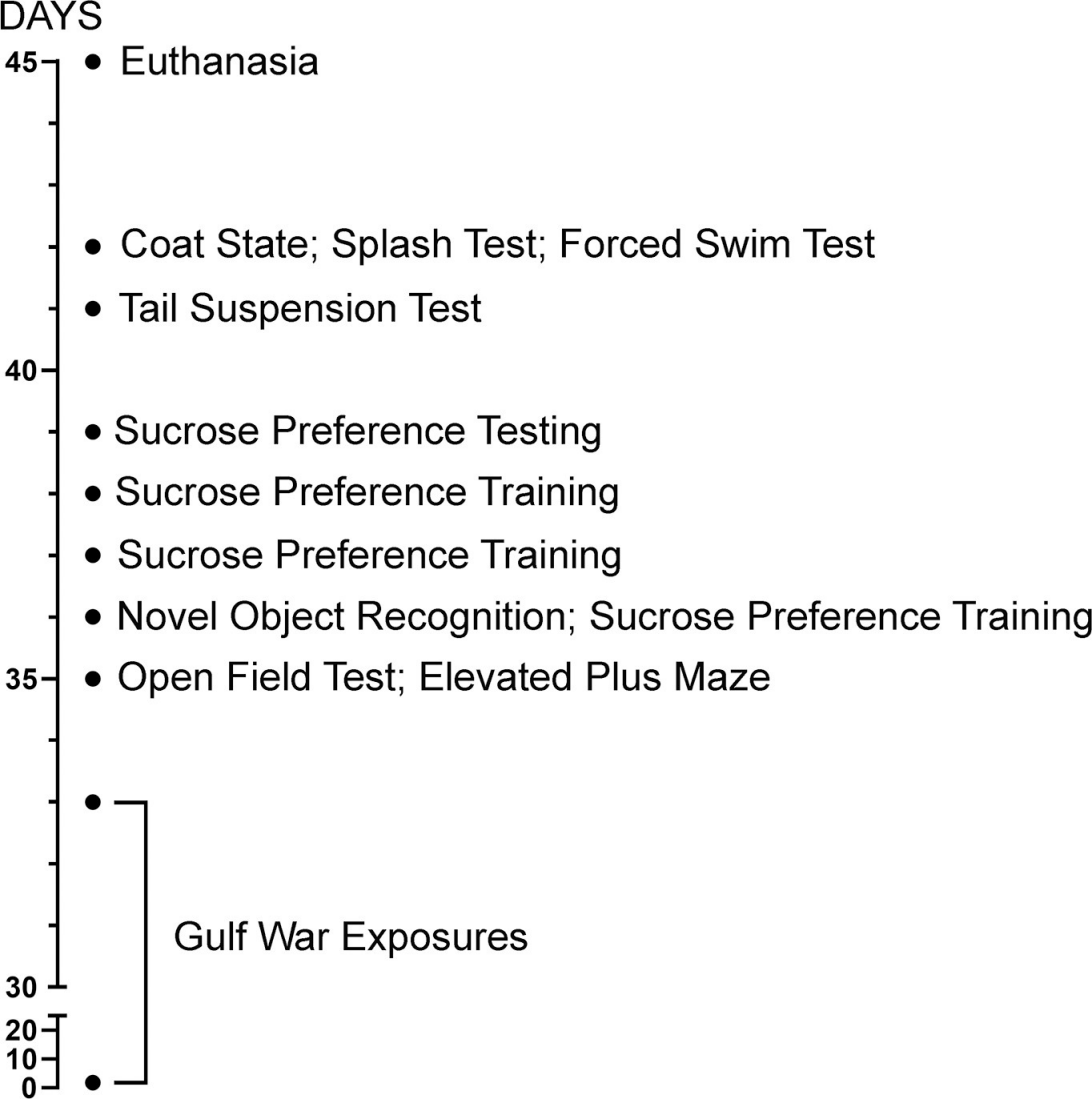
Experimental sequence followed for each of the 3 series of rats studied.

All rats underwent neurofunctional testing beginning on day 35, after the GWE had terminated (Fig 1). We used a battery of tests to evaluate cognition (novel object recognition), anxiety-like behaviors (open field, elevated plus maze) and depression-like behaviors (coat state, splash test, sucrose preference, tail suspension and forced swim). Tests were recorded by an overhead video camera and recordings were analyzed off-line by a blinded investigator. Prior to each test session, apparatuses were cleaned with a wet detergent towel to remove any olfactory cues.

#### Open field test

Spontaneous exploratory behavior was measured in the open-field test, which was performed on day 35 as described [69]. Rats were placed individually in an open field (16×16×14 inches) to explore freely for 5 minutes per session. Time spent in the center and vertical activity (rearing time) were analyzed.

#### Elevated plus maze

The elevated plus maze, performed on day 35, was used to assess anxiety-like behavior, as described [70]. The apparatus (Coulbourn Instruments, Holliston, MA) consists of two open (50×10 cm) and two closed (50×10 cm) arms. The closed arms are surrounded by 30 cm high walls painted black. Open and closed arms enclosed a central region of 10×10 cm. The maze was elevated to a height of 50 cm from the floor. The rat was placed on the center of the maze with its face towards the open arm. Behavior was recorded for a period of 5 minutes, and time spent in the open arms was determined. A rat was considered to be in an open arm if its head and body to at least the forelimbs was on in an open arm.

#### Novel object recognition

The novel object recognition test was performed on day 36as described [71] using the same field as the open field test. The objects used were of similar size, made of easy-to-clean plastic materials. During the acquisition phase, two identical objects were set in the field at a distance from each other. The animal placed in the testing arena was allowed to explore freely for 5 minutes and was then returned to its home cage. The objects were replaced by another set of objects, one identical and one novel; after 20 minutes, the animal was returned to the arena and allowed to explore the objects for 5 minutes (recognition phase). The time spent interacting with each object as well as the overall time exploring the objects, whether old and new (total exploration time) were measured. The recognition index was calculated as follows: time spent interacting with the novel object × 100 / total exploration time. Animals able to discriminate the new from the old object should have a recognition index greater than 50%.

#### Sucrose preference training and testing

Anhedonia, measured as reduced preference for sweet solutions constitutes, with social avoidance or isolation, a feature closely related to the state of defeat in animals [72]. The sucrose preference training was performed from day 35 to 38 as described [73], with minor modifications. On the first day, animals were free to drink from two bottles of 1% sucrose water. The next day, one of the sucrose water bottles was replaced by one with ordinary distilled water. After 22 hours of fasting, the animals were given one bottle of 1% sucrose and the other bottle of normal tap water. Baseline weight of the bottles were recorded. After 1 hour, the consumption was calculated by weighing the bottles. The sucrose preference was calculated as follows: sucrose preference = sucrose consumption / (water consumption + sucrose consumption) × 100%.

#### Tail suspension test

The tail suspension test is a behavioral despair model of depression used in anti-depression studies of rodents [74]. On day 41, rats individually were suspended 58 cm above the floor by adhesive tape 3 cm wide, placed approximately 2 cm from tip of the tail. A platform with soft covering was placed horizontally 10–20 cm (depending on the animal’s size) just under the rat’s forepaw. Rats were considered immobile only when they hung passively and motionless. The mobility was recorded and was subtracted from 6 minutes (duration of the test) in order to get immobility time.

#### Coat state

Dirty coats in rats often represent low self-care behavior (e.g., unwillingness to self-clean). On day 42, the observed coat state was evaluated with a quantitative scale that assessed eight different body parts: head, neck, dorsal coat, tail, forelimb, hindlimb, ventral coat, and genital region [67]. The total score of the coat status was obtained by summing up the scores for each individual part either 0 (clean coat) or 1 (dirty coat or in abnormal state) to each of the eight parts.

#### Splash test

The splash test was performed in all groups just before the forced swim test at day 42. An atomizer spray containing 10% sucrose solution was sprayed on the dorsal coat of a rat in its home cage [67]. After applying sucrose solution, the latency to start grooming and the time spent grooming was recorded for a period of 5 minutes as an index of self-care and motivational behavior [75, 76]. The proportion of time spent in grooming (= grooming time / 5 min) was calculated.

#### Forced swim test

On day 42, rats were forced to swim and the swimming was recorded. Rats were individually placed into a rectangular container (60 × 30 × 40 cm) containing 30 cm of water at room temperature. The animals were left to swim for 6-minutes, during which the following behavioral responses were recorded: (1) immobility (time spent floating with the minimal movements to keep the head above the water) and (2) swimming (time spent with active swimming movements). The mobility time was calculated at the last 4 minutes of the 6-minute swim, and the immobility time was calculated by subtracting mobility time (sec) from total time (240 sec.).

### Immunohistochemistry

After neurofunctional assessments were completed, rats were euthanized using an overdose of sodium pentobarbital (>100 mg/kg), and brain tissues were harvested. Brains from naïve rats in *series 1* and both groups in *series 3* were used for immunohistochemistry, which was performed as described [77]. Rats were euthanized and transcardially perfused with normal saline and 10% buffered formalin. Brains were quickly removed, placed in 10% formalin for 24 h at 4°C, cryo-protected with 30% sucrose for 48 h, and embedded in Tissue-Tek^®^ O.C.T.™ Compound (Sakura Finetek, Torrance, CA). Coronal cryosections (10 μm), collected between 2.5 and 3 mm caudal to bregma, were mounted on slides, blocked with 2% donkey serum with 0.2% Triton X-100, and incubated overnight with primary antibodies directed against: glial fibrillary acidic protein (GFAP) (Cy3-conjugated; 1:500; Sigma, St. Louis, MO), ionized calcium-binding adapter molecule 1 (Iba1) (cat# 019–19741; 1:200; Wako Chemicals, USA, Richmond, VA) tumor necrosis factor (TNF) (cat# sc1350; 1:200; Santa Cruz Biotechnology, Santa Cruz, CA) and translocator protein (TSPO) (cat# PA5-75544; 1:100; Invitrogen, Carlsbad, CA). Species appropriate Alexa Fluor 500- or fluorescein isothiocyanate (FITC)-conjugated secondary antibodies were applied, and tissues were cover-slipped with ProLong Gold antifade reagent containing 4′,6-diamidino-2-phenylindole (DAPI) (cat# 8961S; Cell Signaling Technologies, Danvers, MA). Controls included omission of primary antibody.

Unbiased measurements of specific labeling within regions of interest (ROIs) were obtained using NISElements AR software (Nikon Instruments, Melville, NY) from sections immunolabeled in a single batch. All images for a given signal were captured using uniform parameters of magnification, area, exposure, and gain. Segmentation analysis was performed by computing a histogram of pixel intensity for a particular ROI, and pixels were classified as having specific labeling, based on signal intensity greater than 2× that of background. The area occupied by pixels with specific labeling was used to determine the percent area in the ROI with specific labeling (% ROI). For GFAP and Iba1 in the hippocampus, the ROI (2000×1000 μm) was positioned at hilus of dentate gyrus. For GFAP and Iba1 in the amygdala, the ROI (1000×1000 μm) was positioned at the lateral amygdala. For TNF and TSPO, ROIs were defined as pixels with specific labeling for GFAP or Iba1 within the ROI defined above for hippocampus and amygdala.

### Data analysis

Statistical analyses were performed using GraphPad Prism 8.3 (San Diego, CA). A one-way ANOVA with Tukey’s post-hoc comparisons or Kruskal-Wallis test with Dunn’s post-hoc comparisons was used as appropriate. A value of *p* < 0.05 was considered statistically significant.

A preliminary analysis was carried out to determine whether there were any differences in the 10 measures from the 8 neurofunctional tests when comparing 4 groups: male rats from *series* 1, 2 and 3 and female rats from *series* 2, all with GWE without treatment. An ANOVA for 9 of the tests revealed no significant differences (*p* > 0.05). The single test that showed significance (*p* < 0.05) was the open field–time rearing test, with post-hoc analysis showing a difference between male rats with GWE from *series* 1 and *series* 3, due to 3 extreme data points from *series* 1; these 3 data points did not qualify as outliers (ROUT method in GraphPad) and so they were retained. Given the results of this preliminary analysis, the data from these 4 groups were pooled as the GWE group. Thus, 3 groups were submitted to final analysis: (i) naïve (n = 14); (ii) GWE (n = 36); (iv) GWE-ROSI (n = 10).

Nominal data are presented as mean ± 95% confidence interval (neurofunctional data) or as mean ± S.D. (serial weights) or mean ± S.E. (immunohistochemistry).

## Results

### Body mass

**Fig 2:**
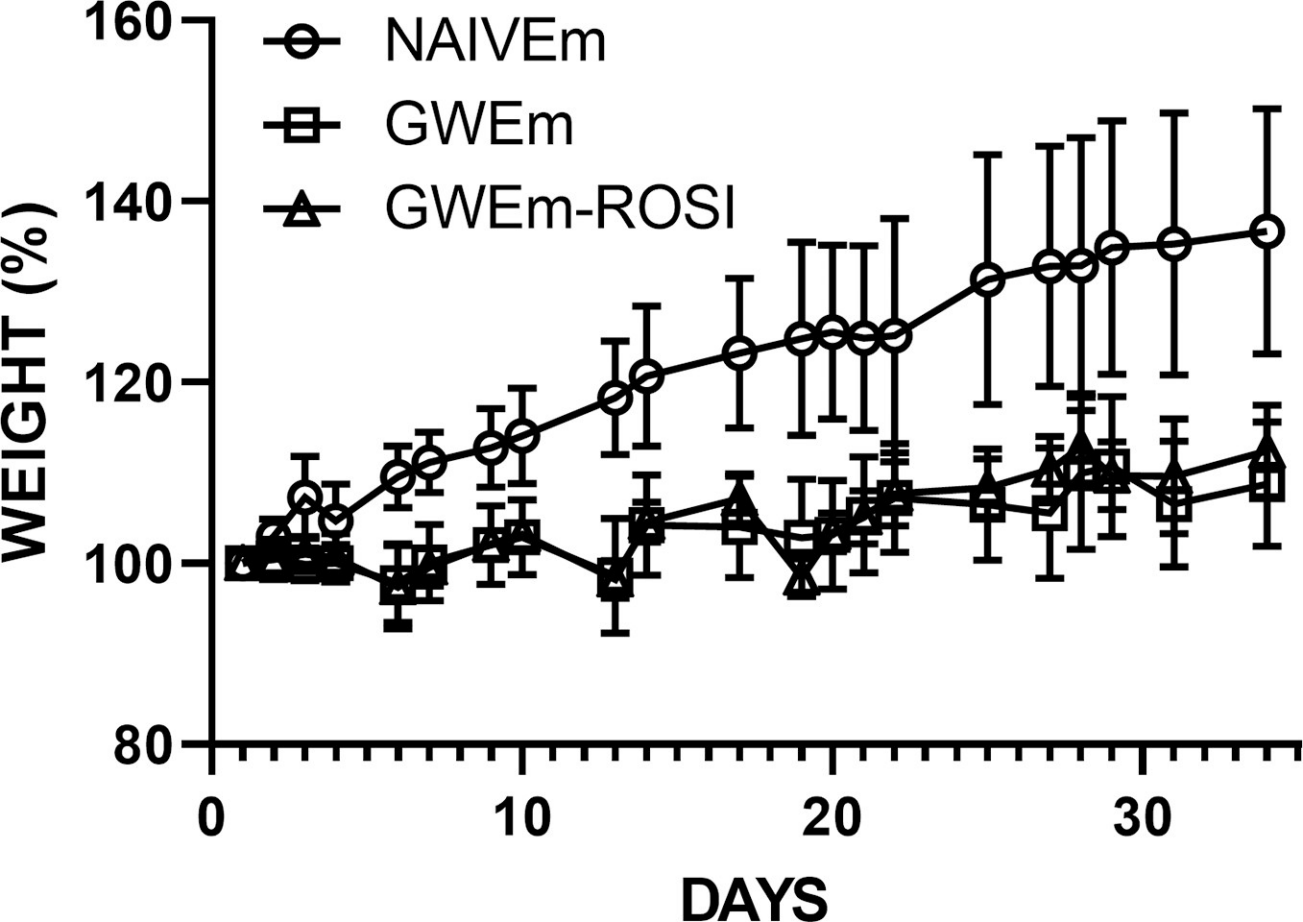
Normalized weight (mean ± SE) as a function of time in naïve male rats (NAIVEm) vs. male rats with the 33-day GWE (GWEm), including those with rosiglitazone (GWEm-ROSI).

There were no differences in body weight among males at the beginning, but significant differences emerged during the course of the 33-day exposures. Compared to naïve controls, males with GWE gained body weight more slowly (Fig 2). Daily observation revealed no signs of overt toxicity, inflammation, sepsis or seizures. There was no mortality in any of the groups with GWE.

### Neurofunction

At the end of the 33-day GWE, starting on Day 35 and over the course of 1 week, rats underwent a battery of tests to evaluate three domains: cognition (novel object recognition), anxiety-like behaviors (open field, elevated plus maze) and depression-like behaviors (coat state, splash test, sucrose preference, tail suspension and forced swim).

In a preliminary analysis, no statistically significant differences were found for any of the 10 measures from the 8 neurofunctional tests between male and female rats with GWE–females were as severely affected as males, compared to naïve. Because outcomes were not distinguishable, we pooled the data from GWE rats regardless of sex. This left 3 groups for comparison: naïve, GWE and GWE-ROSI.

**Fig 3:**
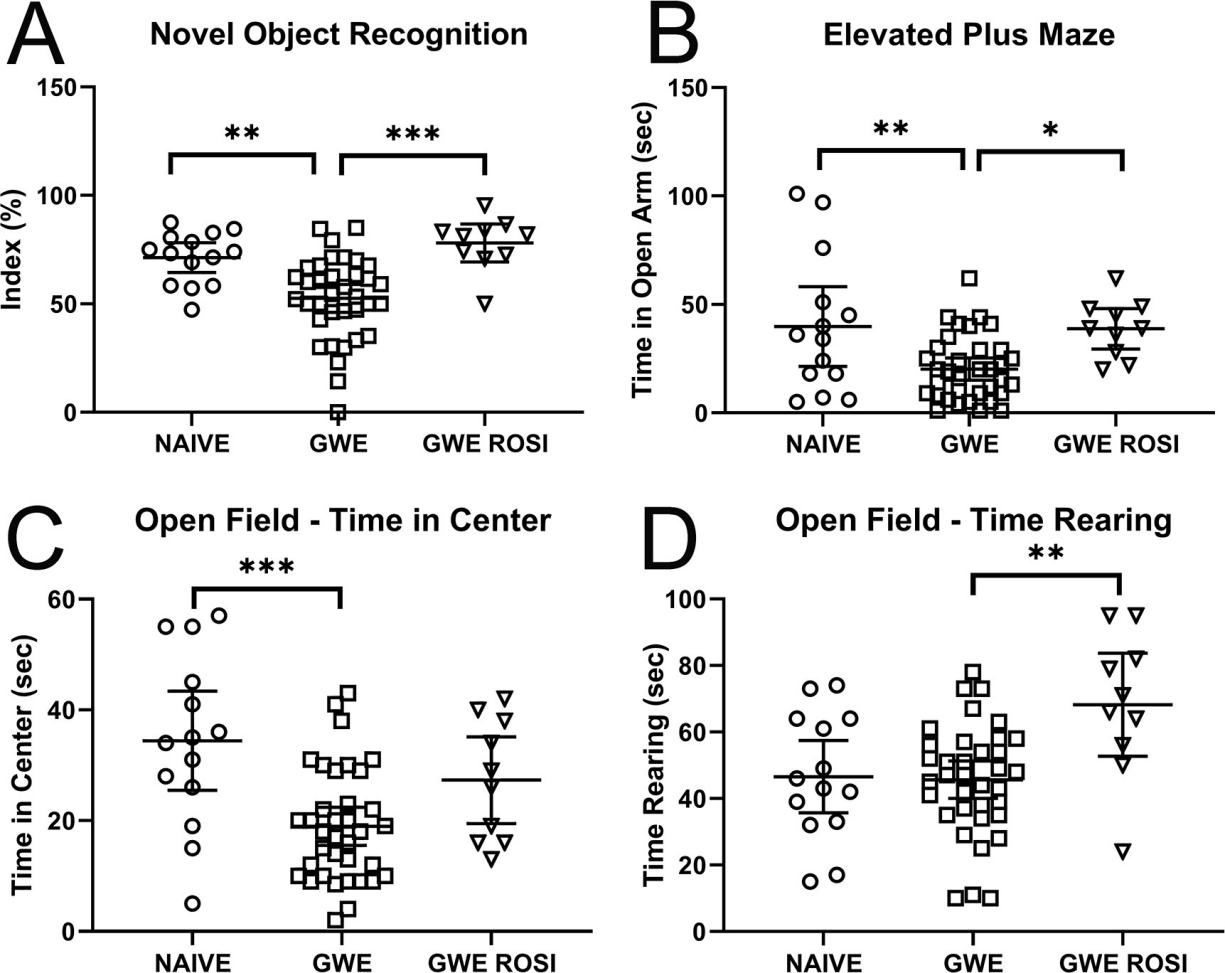
Neurofunctional tests for cognition and anxiety-like behaviors. *, *p* < 0.05; **, *p* < 0.01; ***, *p* < 0.001.

**Fig 4:**
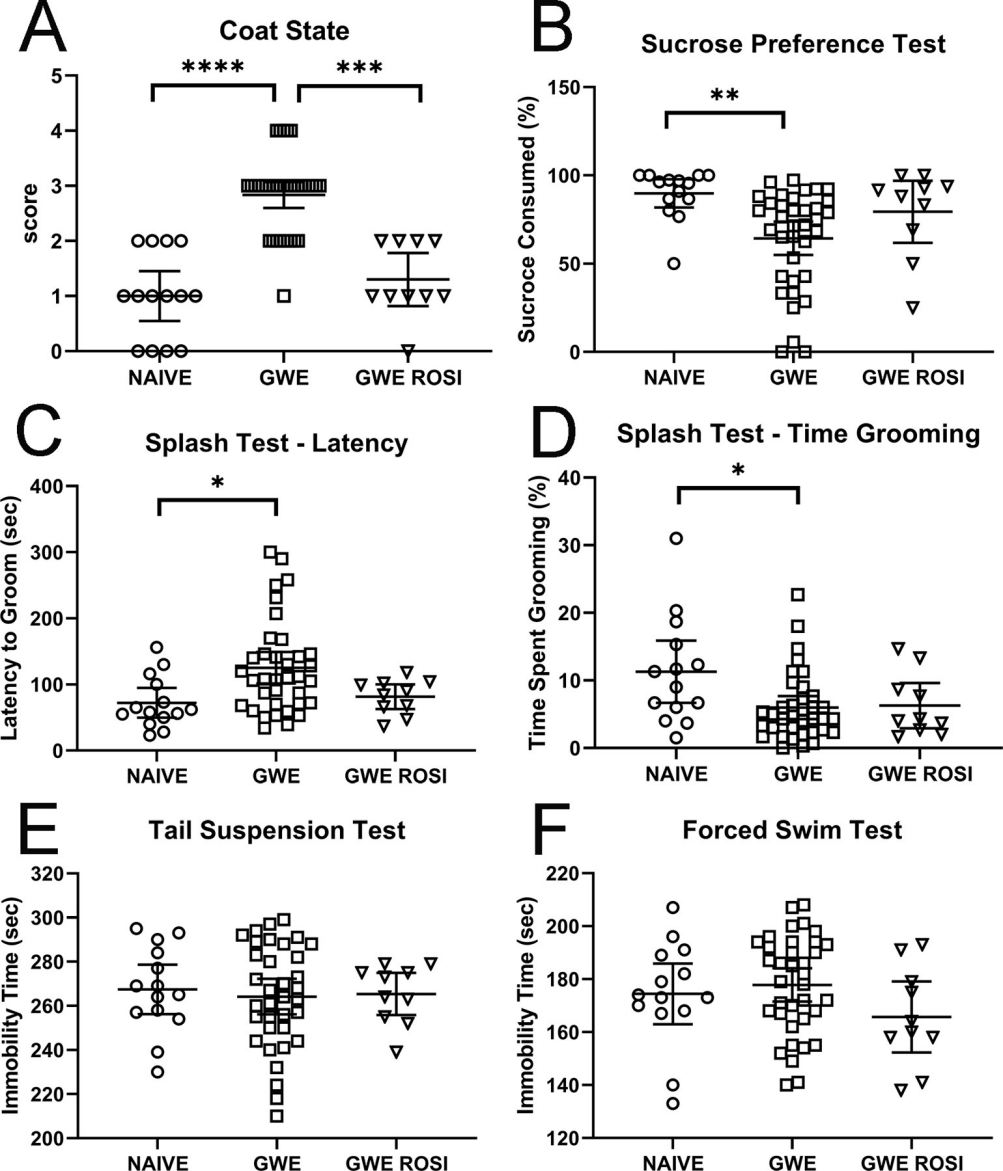
Neurofunctional tests for depression-like behaviors. *, *p* < 0.05; **, *p* < 0.01; ***, *p* < 0.001; ****, *p* < 0.0001.

We first compared neurofunctional data from rats with GWE vs. naïve rats. With GWE, abnormalities were detected in all three domains, although not with all tests. GWE lead to a significant reduction in the cognitive test, novel object recognition (Fig 3A). GWE also resulted in alterations in anxiety-like measures, including significant reductions in the time spent in both the open arm of the elevated plus maze and in the center of the open field (Fig 3B and 3C). However, time spent rearing in the open field was not affected by GWE (Fig 3D). The results with tests for depression-like behaviors were mixed. GWE led to a significant increase in the score for the coat state, a significant reduction in sucrose preference, and significant changes in the splash test, both the latency and the time spent grooming (Fig 4A–4D). However, both the tail suspension test and the forced swim test revealed no effect of GWE.

A second objective was to evaluate the effect of prophylactic treatment with rosiglitazone administered during the GWE. For this experiment, prophylactic treatment was administered beginning at the start of the GWE and continued daily for 33 days. Because rosiglitazone was administered *per orem*, it was made palatable by mixing with maple syrup, as described [63], necessitating that control rats that were run at the same time (*series* 3) receive the same vehicle. Preliminary analysis revealed no statistically significant differences in any of the 10 neurofunctional tests between rats with GWE receiving vehicle vs. those without vehicle.

We compared neurofunctional data from rats with GWE vs. GWE-ROSI. A benefit of ROSI was detected in all three domains, although not with all tests. In the cognitive test of novel object recognition, ROSI fully reversed the effect of GWE (Fig 3A). In the measures of anxiety-like behaviors, ROSI reversed the reduction in time spent in the open arm of the elevated plus maze induced by GWE, but not the time spent in the center of the open field (Fig 3B and 3C). ROSI increased the time spent rearing in the open field above that observed in both the naïve and the GWE groups (Fig 3D), but the meaning of this is unclear. The results with tests for depression-like behaviors were less favorable. Only the score of the coat state was returned to near-normal by ROSI, whereas ROSI did not significantly improve sucrose preference or the splash test latency or time spent grooming compared to GWE (Fig 4A–4D). Both the tail suspension test and the forced swim test were unaffected by ROSI.

### Neuroinflammation

**Fig 5:**
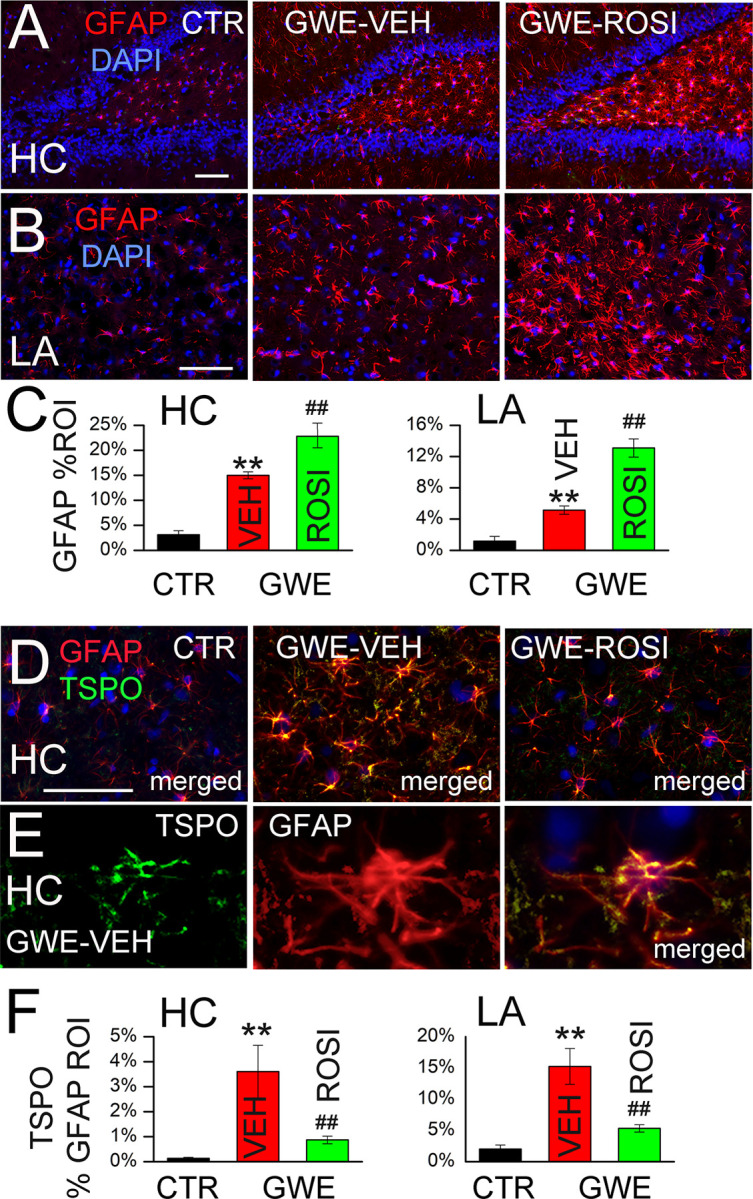
Astrocytosis following 33-day GWE. **A,B:** Immunolabeling for GFAP in hippocampus (HC) and lateral amygdala (LA) in naïve (CTR), GWE-VEH and GWE-ROSI rats; nuclear staining with DAPI (blue) is also shown; *bars*, 100 μm. **C:** Quantification of GFAP in the two brain regions for the three groups, as indicated; 5 rats per group; **, *P*<0.01, compared to naïve; ##, *P*<0.01, compared to GWE-VEH. **D:** Co-immunolabeling for GFAP and TSPO in hippocampus in naïve (CTR), GWE-VEH and GWE-ROSI rats; nuclear staining with DAPI (blue) is also shown. **E:** High magnification image of a cell co-immunolabeled for GFAP and TSPO, showing TSPO within the GFAP-positive cell. **F:** Quantification of TSPO within the GFAP-defined ROI in the two brain regions (HC and LA) for the three groups, as indicated; 3–5 rats per group.

We studied the response of astrocytes in the hippocampus (dentate hilus [44, 78]) and lateral amygdala in rats from *series 3* (GWE-VEH and GWE-ROSI), compared to naïve control rats from *series 1*. In both brain regions, GWE led to robust upregulation of GFAP (Fig 5A–5C). Notably, administration of ROSI resulted in enhanced GFAP expression (Fig 5A–5C), as previously reported [79].

To clarify the seemingly paradoxical GFAP response with ROSI, we examined translocator protein (TSPO), an 18-kDa mitochondrial protein that is upregulated by astrocytes and microglia during neuroinflammatory responses, and thus acts as a sensitive marker of glial activation [80, 81]. As with GFAP, TSPO was markedly upregulated in astrocytes of both brain regions following GWE (Fig 5D and 5F). At high magnification, TSPO was seen to colocalize within GFAP-positive cells (Fig 5E), which allowed using the GFAP ROI to quantify astrocyte-specific expression of TSPO (Fig 5F). Unlike GFAP, administration of ROSI yielded a significant reduction in TSPO (Fig 5D and 5F), consistent with a reduction in the inflammatory response.

**Fig 6:**
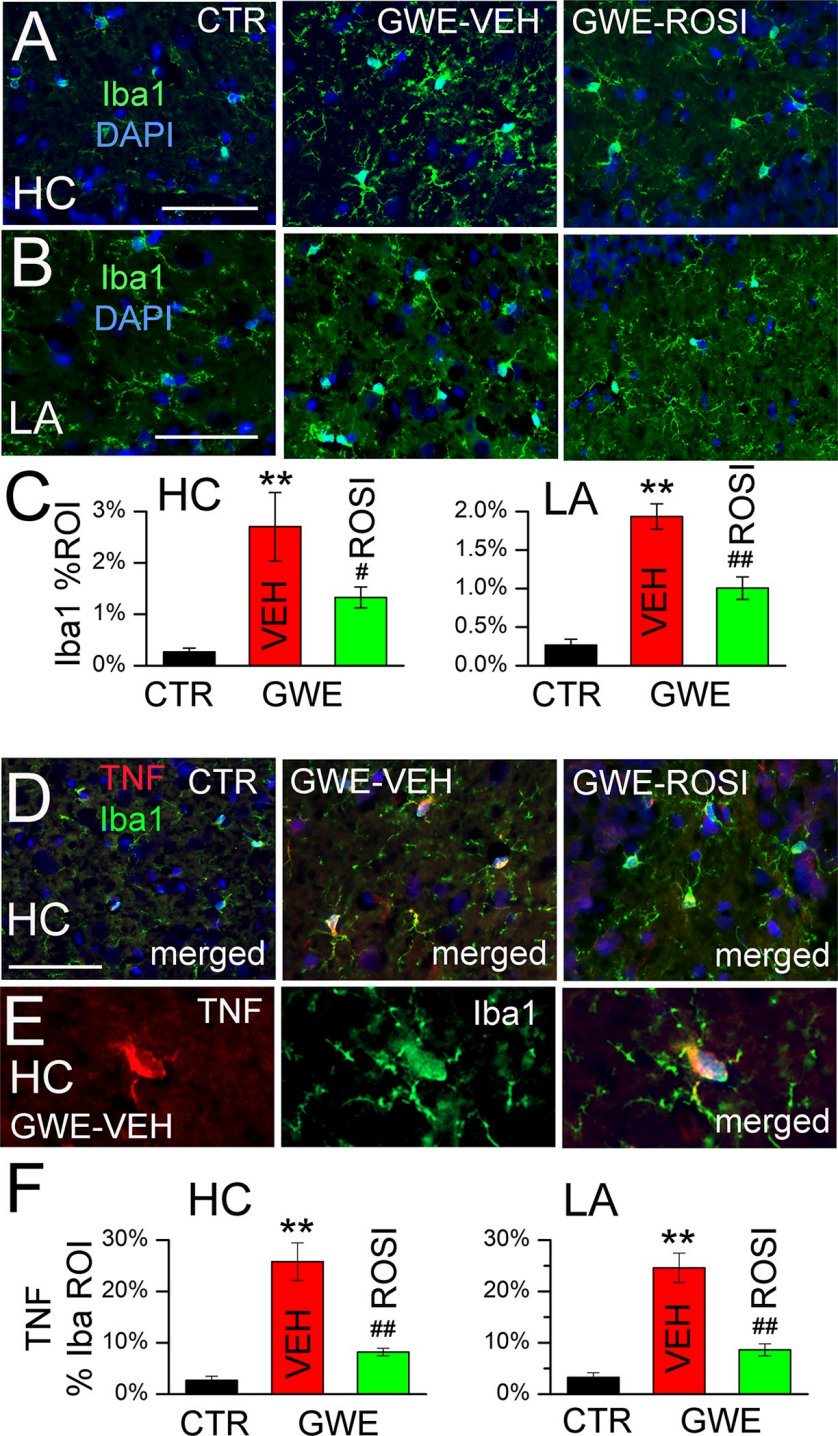
Microglial activation following 33-day GWE. **A,B:** Immunolabeling for Iba1 in hippocampus (HC) and lateral amygdala (LA) in naïve (CTR), GWE-VEH and GWE-ROSI rats; nuclear staining with DAPI (blue) is also shown; *bars*, 100 μm. **C:** Quantification of Iba1 in the two brain regions for the three groups, as indicated; 5 rats per group; **, *P*<0.01, compared to naïve; #, *P*<0.05 and ##, *P*<0.01, compared to GWE-VEH. **D:** Co-immunolabeling for Iba1 and TNF in hippocampus in naïve (CTR), GWE-VEH and GWE-ROSI rats; nuclear staining with DAPI (blue) is also shown. **E:** High magnification image of a cell co-immunolabeled for Iba1 and TNF, showing TNF within the Iba1-positive cell. **F:** Quantification of TNF within the Iba1-defined ROI in the two brain regions (HC and LA) for the three groups, as indicated; 5 rats per group.

We studied the response of microglia (Iba1) in the hippocampus and lateral amygdala of the same rats. In both regions, GWE led to morphological changes consistent with microglial activation, including robust upregulation of Iba1 (Fig 6A–6C). Administration of ROSI significantly attenuated these responses (Fig 6A–6C).

The microglial response to GWE was accompanied by commensurate responses in TNF expression (Fig 6D). At high magnification, TNF was seen to colocalize within Iba1-positive cells (Fig 6E), which allowed using the Iba1 ROI to quantify microglia-specific expression of TNF (Fig 6F). Administration of ROSI yielded a significant reduction in TNF (Fig 6D and 6F), consistent with a reduction in the inflammatory response.

As with the astrocyte responses, TSPO also was upregulated in microglia of both brain regions following GWE, and administration of ROSI yielded significant reductions in TSPO (not shown).

## Discussion

GWI remains a complex, untreatable illness for up to 250,000 veterans of the Persian Gulf War, with cognitive dysfunction and mood deficits being among the most prevalent and debilitating symptoms. Although the etiology of GWI is unknown, epidemiological investigations and experimental studies using animal models indicate that these impairments are likely related to chemical exposures that were encountered during deployment [1, 2, 36, 82, 83]. It has been hypothesized that various combinations of chemicals together with the stress encountered in theater may have contributed collectively and synergistically to induce GWI [1, 2, 35, 36].

In this study, we sought to explore a model of GWI based on plausible exposures that would induce neurofunctional and neuroinflammatory abnormalities in rats mimicking those in Veterans with GWI. Although many chemical and other exposures have been identified as potentially contributing to GWI, in our model we included pyridostigmine and chronic unpredictable stress, based on the report that, among personnel who were in Iraq or Kuwait, GWI was most strongly associated with using pyridostigmine bromide pills and being within one mile of an exploding SCUD missile [36]. To this pair of exposures, we added a third one, intranasal LPS, since this was an almost inescapable exposure due to frequent sandstorms in Iraq and Kuwait, and since desert sand from the Middle East is reported to be especially rich in LPS [33]. Inclusion of intranasal LPS is a novel aspect of GWI modeling.

Several aspects of our model, including the incorporation of stress and use of the intranasal route for LPS administration, likely contributed in unique ways to the overall effects that we observed. The intranasal route of drug administration is considered to be a non-invasive alternative route to bypass the blood-brain barrier (BBB) and directly target the CNS [84]. Intranasal drug administration facilitates drug entry into the brain via the olfactory apparatus, with the olfactory nerves projecting directly to the limbic system, including the amygdala. Moreover, there is a critical interaction between exposures to agents unique to this war and the stress that was incumbent within the GW theater. This critical issue was first reported experimentally by O’Callaghan et al. [38], who elegantly reaffirmed the epidemiological observations of Steele et al. regarding pyridostigmine and SCUD missiles [36]. Stress is known to compromise the BBB, thereby facilitating passage into the brain of chemicals such as PYR and possibly LPS that might otherwise be largely excluded [85]. Notably, the harmful effects of stress on the BBB may be especially prominent in the amygdala and hippocampus [86], the two areas that we focused on due to their importance in emotional and cognitive functions.

Our model with three GWE lasting 33 days yielded significant neurofunctional abnormalities, as have other models of GWI [18, 21, 45, 48]. We used an extensive battery of tests to evaluate three neurofunctional domains: cognition, anxiety and depression. Comparing GWE-rats vs. naïve rats, GWE led to significant abnormalities in all three domains, in both males and females. Comparing naïve vs. GWE, several measures acted as robust discriminators, including novel object recognition, elevated plus maze, open field-time in center, coat state and sucrose preference; the splash test, both latency and time grooming also showed significance, but was less robust. In the case of depression-like behaviors, whereas the coat state, sucrose preference and splash test were abnormal, tail suspension and forced swim were not. The forced swim test, widely used for detecting antidepressant drug activity in rats [87], has been criticized as having relatively little validity to support an interpretation of "depression-like" behavior, and may instead be a reflection of coping strategy (active vs. passive coping) for an acute inescapable stress [88, 89]. The same has been said of the tail suspension test [90]. Overall, our findings indicate that the model with three GWE lasting 33 days yielded significant, clinically relevant neurofunctional abnormalities that are consistent with major features of GWI in humans.

Our model with three GWE lasting 33 days also yielded significant neuroinflammation, as have other models of GWI [16–18, 22, 38, 39, 44, 47, 48, 50]. Our focus was on amygdala and hippocampus, because of their importance for memory and complex emotive function. Several previous studies on GW models reported neuroinflammatory changes in the hippocampus [24, 40, 41, 46, 47, 62], whereas fewer studies examined the amygdala [17, 40]. In both regions, we found that GWE led to significant astrogliosis with GFAP upregulation, as well as microgliosis, with bushy, ameboid-appearing Iba1 positive microglia. These changes in cellular activation were accompanied by upregulation of TNF, as has been found in other models of GWI [46, 50, 91].

We used TSPO expression to corroborate neuroinflammation in our model with GWE. TSPO is an18-kDa mitochondrial protein that is constitutively expressed at low levels by many cell types within the healthy CNS [92, 93], but is markedly upregulated by microglia/macrophages and astrocytes during neuroinflammatory responses [80, 81]. TSPO thus acts as a sensitive marker of glial activation. Here, in the first report to examine TSPO in an animal model of GWI, we found that TSPO was significantly upregulated, and that it colocalized with both astrocytes (GFAP) and microglia (Iba1) in amygdala and hippocampus. Recent work using PET imaging with the TSPO ligand, PBR28, provided the first evidence of active neuroinflammation *in vivo* in Veterans with GWI [53]. This recent report in humans, together with our findings in rats, provides a unique link between animal model and human disease.

Previous work has shown that treatments to reduce neuroinflammation, including oleoylethanolamide [22], curcumin [48] and monosodium luminol [51], improve neurofunctional abnormalities in GWI models. To date, however, effects of PPARγ agonists have not been investigated. Here, we found that prophylactic treatment with the PPARγ agonist, rosiglitazone, was effective in ameliorating both neuroinflammatory and certain neurofunctional abnormalities arising from the GWE. The neurofunctional manifestations of GWE that were best treated by ROSI were those related to cognition and anxiety. By contrast, weight gain and most measures of depression-like behaviors in GWE-rats were not significantly affected by ROSI. The effects on neuroinflammation are in agreement with earlier findings that rosiglitazone, apart from its antihyperglycemic property, also exerts anti-neuroinflammatory effects in various models of CNS injury [57, 94]. The mechanisms underlying the effects of PPAR-γ agonists in ameliorating inflammation are not completely understood. Most of the reports on the mechanism of action of PPAR-γ agonists are based on studies of NF-κB activity. PPAR-γ agonists inhibit multiple steps in the NF-κB signaling pathway through covalent modifications of IκB kinase [95–97] and transrepression of NF-κB, resulting in suppression of inflammation [98, 99]. However, it has also been reported that anti-inflammatory actions of PPAR-γ agonists in activated glial cells may be based on the JAK-STAT signaling pathway [100]. Further work will be required to elucidate the relative contribution of these mechanisms in the salutary effects observed with rosiglitazone in our model of GWI.

## Conclusion

The work presented here shows that a novel model of GWI involving three plausible exposures recapitulates important CNS manifestations of GWI in male and female rats in the domains of cognition, anxiety and depression. This model was characterized by neuroinflammation in hippocampus and lateral amygdala, two brain regions critically involved in cognitive and emotive functions. Neuroinflammation was characterized by TSPO upregulation in astrocytes and microglia, similar to observations in Veterans with GWI. Our studies show that prophylactic treatment with rosiglitazone during the GWE significantly ameliorated both neuroinflammatory and certain neurofunctional abnormalities. A cure for GWI is not yet at hand, but work by several groups including ours suggests that the harmful effects of similar in-field exposures in the future may possibly be lessened by prophylactic treatment targeting neuroinflammation.

## Supporting information

S1 Data (XLSX)
